# Perils of the Restaurant

**Published:** 1920-09-11

**Authors:** 


### Perils of the Restaurant.

### THE DECLINE OF HOME COOKING.

An important and interesting question with regard to restaurants and eating-houses is raised by Dr. Charles Porter, the medical officer of health for Marylebone, in his annual report for 1919. For a variety of reasons the number of premises in St. Marylebone in which meals are provided or food is sold ready cooked or is prepared for sale is very large, and include restaurant, dining-room and coffee-shop kitchens, tea-rooms and pastry-cooks, hotel kitchens, fried-fish shops, and fish-curers. In addition, there are a number of shops in which meat, ham, sausages, etc., are cooked and sold only over the counter.

None of these premises, he says, is, or is required to be, registered, and apart from fish-curing and fish-frying premises, which are required to be in compliance with certain requirements contained in by-laws of the London County Council, none of them is subject to any very special provisions. Any individual, in short, may take any sort of premises, and proceed to cook and prepare food for sale; no notice to the local authority is required, and unless complaint is received, or infectious or suspicious illness occurs, or the premises are visited by an inspector, the fact that they are so used may never be discovered.

That this is so appears to be at least unsatisfactory. On account of changed conditions the amount of home cooking done is steadily diminishing, and more and more the cook-shop, the restaurant, and the cooked-food shop are being resorted to. In the poorer quarters of the borough the extent to which the fried-fish shop, for example, is depended upon may be judged from the number of children making purchases during the school dinner-hour, and the number of adult and child customers in the evenings. In the evenings, too, the busiest shops in the main thoroughfares are those where cooked foods are retailed over the counter.

Legislation, which did not foresee these changes, contains no provision for dealing properly with the premises, and the Legislature has made no attempt to obtain control over them. Such places as the law, as it now stands, requires to be registered or licensed are mainly those in which uncooked food is dealt with, *e.g.*, slaughter-houses, cow-sheds, milk-shops, etc.

Having regard to the changes the law should be extended in order to provide for registration of the cooked-food premises also. Before establishing a restaurant or eating-rooms of any description, a cook-shop or a cooked-food shop, it should be necessary for application for registration to be made to the local authority, and registration should not be granted until the premises had been inspected and passed as suitable.

